# Pericardial effusion in hypothyroidism: A case report

**DOI:** 10.1016/j.amsu.2021.102999

**Published:** 2021-10-30

**Authors:** Saurab Karki, Rabindra Jang Rayamajhi, Shreeja Shikhrakar, Sunil Shahi, Binaya Dhakal, Manoj Khadka

**Affiliations:** a Military Hospital, Sunsari, Nepal; b Department of Internal Medicine, Shree Birendra Hospital, Kathmandu, Nepal; c Kathmandu University School of Medical Sciences, Dhulikhel, Nepal; d Military Hospital, Banke, Nepal; e Nepalese Army Institute of Health Sciences, Kathmandu, Nepal

**Keywords:** Case report, Hoarseness, Hypothyroidism, Pericardial effusion

## Abstract

**Introduction:**

and importance: Hypothyroidism is an endocrine disorder with multiorgan involvement and various complications. One of the significant but less often seen complications is pericardial effusion. Since it can progress to life-threatening conditions like cardiac tamponade and hemodynamic instability, early diagnosis, and management of the pericardial effusion in hypothyroidism is a must.

**Case presentation:**

We present a case of a 35-year-old male who presented with bilateral lower limb swelling, facial puffiness, cold intolerance, fatigue, and hoarseness of voice for one week. Laboratory investigation showed high thyroid-stimulating hormone (TSH), low triiodothyronine (T3), and raised serum anti-thyroid peroxidase (anti-TPO). The lipid profile demonstrated hypertriglyceridemia. Ultrasonography of the neck revealed normal thyroid size with decreased echo texture and increased vascularity. An electrocardiogram showed low voltage complexes with sinus bradycardia. 2D echocardiography revealed minimal pericardial effusion with normal ventricular function. The patient was managed with thyroxine therapy which gradually resolved his symptoms and pericardial effusion.

**Clinical discussion:**

Pericardial effusion in hypothyroidism is due to the increased capillary permeability and albumin distribution volume and reduced lymph drainage in the pericardial cavity. Its presence in mild cases of hypothyroidism is uncommon although it can be seen in severe, long-standing hypothyroidism. Pericardial effusion in hypothyroidism, though rare, can present in mild cases and if overlooked can be fatal due to conditions like cardiac tamponade.

**Conclusion:**

With early cardiac assessment and adequate thyroid replacement therapy, pericardial effusion in hypothyroidism can be reversible at an early stage. So, pericardial effusion which can be overlooked in mild cases of hypothyroidism needs to be identified and managed early.

## Highlights

• Hypothyroidism is an endocrine disorder with multiorgan involvement.
• Pericardial effusion is a notable complication of hypothyroidism, occurring due to the increased capillary permeability and albumin distribution volume and reduced lymph drainage in the pericardial cavity.
• The incidence of pericardial effusion is higher in severe, long-standing hypothyroidism whereas it’s uncommon in early stages and mild form of hypothyroidism.
• If presents earlier, with early cardiac assessment and adequate treatment, pericardial effusion can be reversible at an early stage.

## 1 Introduction

Hypothyroidism is a prevalent endocrine disorder with a wide range of symptoms and multiorgan involvement [1]. Among its numerous complications, pericardial effusion is a notable complication [2]. Pericardial effusion can occur in severe and long-standing hypothyroidism although it is uncommon in mild cases and can rarely present even as the primary presentation of hypothyroidism [3,4].

Herein we report a case of a 35-year-old male with hypothyroidism and minimal pericardial effusion. This study shows that pericardial effusion can occur at initial presentation in mild cases too and with early cardiac assessment and adequate thyroid replacement therapy, pericardial effusion in hypothyroidism can be reversed at an early stage. This case report has been reported in line with the SCARE 2020 criteria [5].

## 2 Case presentation

Thirty-five years male non-smoker and non-alcoholic with no known comorbidities presented to our institution with complaints of bilateral lower limb swelling of one-week duration. The swelling was insidious in onset and gradually progressive. It initially started from the lower leg and progressively moved upward. It was associated with facial puffiness, cold intolerance, fatigue, and hoarseness of voice; however, there was no history of abdominal swelling, shortness of breath, chest pain, palpitation, reduced urine output, or passing frothy or blood in the urine. His bowel and bladder habits were normal. There was no drug history, no surgical history, and no history of similar illness in the past, or on any other family members.

On examination, his pulse was 54 beats per minute, regular, normal in volume. His blood pressure was 110/60 mm of Hg, respiratory rate was 16 per minute, and he was afebrile. He had bilateral pitting edema over lower limbs along with facial puffiness. There was hoarseness of voice. No neck swelling. All the systemic examinations were normal.

**Fig. 1:**
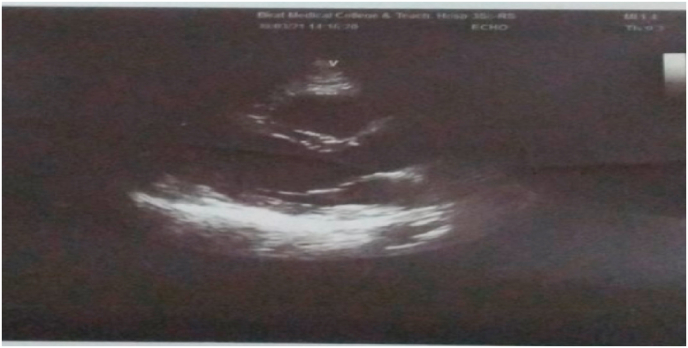
2D echocardiographic findings demonstrating minimal pleural effusion at presentation.

On laboratory investigation, complete blood count (CBC), renal function test (RFT), liver function test (LFT), along total serum protein and albumin were normal. The patient underwent a thyroid function test which revealed high thyroid-stimulating hormone (TSH) > 150 (0.4–6.1) and low triiodothyronine (T3). Ultrasonography (USG) of the neck showed normal thyroid size but decreased echo texture and increased vascularity. Serum anti-TPO antibodies were high of 1412.9 (Negative if < 340/ml). The lipid profile demonstrated hypertriglyceridemia of 435 mg/dl. His serum Vitamin D, calcium, and phosphorus level were within the normal range. Electrocardiogram (ECG) revealed low voltage complexes with sinus bradycardia. 2D echocardiography, when done by a cardiologist, revealed minimal pericardial effusion with normal ventricular function (Fig. 1).

He was admitted to the ward and managed with tablet Thyroxine 200 mg OD for two days and continued with 100 mg OD along with anti-lipid drug, tablet Fenofibrate 160mg PO HS. The patient was symptomatically better after a few days of treatment. The pedal edema gradually subsided, and the hoarseness of voice improved after one week of thyroxin therapy. TSH was repeated after two months of thyroxin therapy and was within the normal limit. He was not symptomatic with pericardial effusion, and gradually the effusion subsided, and 2D echocardiography was normal after 2 months of therapy. Presently the patient is taking tab thyroxine 100mg OD and is on regular follow-up.

## 3 Discussion

Hypothyroidism is a prevalent endocrine disease worldwide. It can be asymptomatic or present with a wide range of non-specific symptoms that can delay the diagnosis even when the diagnosis is straightforward with simple laboratory tests [1]. More so with its complications, which can be life-threatening if not diagnosed and managed in time [3].

Pericardial effusion is one of the significant complications in hypothyroidism. The incidence varies to be as high as 80% in severe, long-standing, and congenital hypothyroidism, and only 3% in early stages and mild form of the disease [6]. The frequency of pericardial effusion in well-established myxedema justifies the need to assess pericardial effusion; however, its occurrence in mild cases and as first presentations cannot be overlooked [4,7].

Among the several known etiologies of pericardial effusion, including malignancy, infection, heart failure, radiation, trauma, connective tissue disorder, iatrogenic cause, and metabolic causes, idiopathic pericarditis, usually of viral origin, remains the common cause of pericardial effusion, whereas metabolic disorder like hypothyroidism is considered unusual [8,9].

The pericardial effusion in hypothyroidism is attributed to the increased capillary permeability and albumin distribution volume and reduced lymph drainage in the pericardial cavity [1,8]. The effusion develops over time due to the accumulation of considerable volume without causing cardiac tamponade and alarming symptoms [1,3]. Thus, the absence of clinical symptoms may underestimate the total volume of effusion and disease severity.

Patients with hypothyroidism tend to have pericardial effusion detected by echocardiography up to 30% [9,10]. Cardiomegaly on chest X-ray can have up to 38% false-positive rate and up to 30% false-negative rate, as demonstrated by Kerber et al. for pericardial effusion [11], while neither Kerber et al. nor Hardisty et al. advised low voltage ECG readings to be specific for pericardial effusion [10,11]. Low voltage QRS complex can occur in hypothyroidism due to multiple reasons, notably advancing patient age, notably advancing patient age, thyroid hormone deficiency itself usually severe, or pericardial effusion [12].

Owing to potential complications related to hypothyroidism, identification and subsequent management are required at an early stage. However, due to infrequent association among them, further exploration and experimentation are required to develop optimum care [13]. Just as other symptoms of hypothyroidism, pericardial effusions can be reversed over time with thyroid hormone replacement [14]. Thus, it is important to assess thyroid function in cases of pericardial effusion and vice versa. Also, early assessment and management of pericardial effusion in hypothyroidism are vital to protect patients from life-threatening conditions like cardiac tamponade and hemodynamic instability [13].

## 4 Conclusion

Hypothyroidism is one of the uncommon etiology causing pericardial effusion. Even though it occurs in long-standing myxedema, it can occur in mild cases too, which when unnoticed can be fatal. Since with early cardiac assessment and adequate thyroid replacement therapy, the pericardial effusion due to hypothyroidism can be reversible, it needs to be identified and managed early.

## Sources of funding

None.

## Ethical approval

N/A.

## Author contribution

Author 1: Led data collection, contributed in writing the case information, and initial draft.

Author 2: Concept of study, revising, and editing the manuscript.

Author 3: Contributed in writing introduction, and discussion.

Author 4: Literature review, revising and editing the manuscript.

Author 5: Literature review, revising and editing the manuscript.

Author 6: Revised and edited the rough draft into final manuscript.

All authors were involved in manuscript drafting and revising, and approved the final version.

## Consent

Written informed consent was obtained from the patient for publication of this case report and accompanying images. A copy of the written consent is available for review by the Editor-in-Chief of this journal on request.

## Registration of research studies

1. Name of the registry: N/A.
2. Unique Identifying number or registration ID: N/A.
3. Hyperlink to your specific registration (must be publicly accessible and will be checked): N/A.

## Guarantor

Saurab Karki, Military Hospital, Itahari-4 Sunsari, Nepal. Email: saurabkarki1010@gmail.com, Phone: +977–9841098336.

## Declaration of competing interest

No conflicts of interest.
